# Acute and Delayed Neuromuscular Alterations Induced by Downhill Running in Trained Trail Runners: Beneficial Effects of High-Pressure Compression Garments

**DOI:** 10.3389/fphys.2018.01627

**Published:** 2018-11-28

**Authors:** Sabine Ehrström, Mathieu Gruet, Marlene Giandolini, Serge Chapuis, Jean-Benoit Morin, Fabrice Vercruyssen

**Affiliations:** ^1^Université Côte d’Azur, LAMHESS, Nice, France; ^2^Université de Toulon, LAMHESS, Toulon, France; ^3^Amer Sports Footwear Innovation and Sport Sciences Lab, Salomon SAS, Annecy, France; ^4^Amer Sports Gear and Apparel Innovation and Sport Sciences Lab, Salomon SAS, Annecy, France

**Keywords:** compression garments, soft-tissue vibrations, muscle fatigue, running economy, muscle damage, downhill running

## Abstract

**Introduction:** The aim of this study was to examine, from a crossover experimental design, whether wearing high-pressure compression garments (CGs) during downhill treadmill running affects soft-tissue vibrations, acute and delayed responses in running economy (RE), neuromuscular function, countermovement jump, and perceived muscle soreness.

**Methods:** Thirteen male trail runners habituated to regular eccentric training performed two separate 40-min downhill running (DHR, –8.5°) sessions while wearing either CGs (15–20 mmHg for quadriceps and calves) or control garments (CON) at a velocity associated with ∼55% of VO_2*max*_, with a set of measurements before (Pre-), after (Post-DHR), and 1 day after (Post-1D). No CGs was used within the recovery phase. Perceived muscle soreness, countermovement jump, and neuromuscular function (central and peripheral components) of knee extensors (KE) and plantar flexors (PF) were assessed. Cardiorespiratory responses (e.g., heart rate, ventilation) and RE, as well as soft-tissue vibrations (root mean square of the resultant acceleration, RMS *A_r_*) for *vastus lateralis* and *gastrocnemius medialis* were evaluated during DHR and in Post-1D.

**Results:** During DHR, mean values in RMS *A_r_* significantly increased over time for the *vastus lateralis* only for the CON condition (+11.6%). RE and cardiorespiratory responses significantly increased (i.e., alteration) over time in both conditions. Post, small to very large central and peripheral alterations were found for KE and PF in both conditions. However, the deficit in voluntary activation (VA) was significantly lower for KE following CGs (–2.4%), compared to CON (–7.9%) conditions. No significant differences in perceived muscle soreness and countermovement jump were observed between conditions whatever the time period. Additionally, in Post-1D, the CGs condition showed reductions in neuromuscular peripheral alterations only for KE (from –4.4 to –7.7%) and perceived muscle soreness scores (–8.3%). No significant differences in cardiorespiratory and RE responses as well as countermovement jump were identified between conditions in Post-1D.

**Discussion:** Wearing high-pressure CGs (notably on KE) during DHR was associated with beneficial effects on soft-tissue vibrations, acute and delayed neuromuscular function, and perceived muscle soreness. The use of CGs during DHR might contribute to the enhanced muscle recovery by exerting an exercise-induced “mechanical protective effect.”

## Introduction

Trail running is characterized by the succession of long uphill and downhill sections in a natural environment (*for review*, see Giandolini et al., 2016b). In trail running races where the distance may vary from short (<42 km) to ultra-long (≥100 km), severe alterations in neuromuscular function were reported with substantial failures in both central and peripheral neuromuscular mechanisms for knee extensors (KE) and plantar flexors (PF) (Easthope et al., 2010; Millet et al., 2003, 2011b; Saugy et al., 2013). In these studies, peripheral muscle fatigue may be greatly associated with exercise-induced muscle damage (EIMD) over repeated and prolonged eccentric muscle actions through downhill sections. Intense and/or prolonged downhill running (DHR) is well known to induce a substantial peripheral fatigue and/or low-frequency fatigue in lower limb muscles, assessed from reduced M-waves amplitudes and a decrease in the ratio between force evoked by low-frequency stimulation (e.g., 10–20 Hz) and force evoked by high-frequency stimulation (50–100 Hz) (Giandolini et al., 2016a; Martin et al., 2004). Cellular mechanisms underpinning peripheral fatigue may be attributed to longer muscle lengths (i.e., overstretched sarcomeres) during eccentric muscle actions over braking phases, leading to myofibrillar damage such as disrupted weaker sarcomeres and/or excitation–contraction coupling failure (Douglas et al., 2017; Proske and Morgan, 2001). Although the contribution of central component to neuromuscular fatigue is less important during DHR, central fatigue assessed by a decline in maximal voluntary activation (VA) (2.5–8.0%) was found for KE and PF following a 30-min treadmill DHR (-20%) and a 6.5 km downhill trail run (∼-16%) (Giandolini et al., 2016a; Martin et al., 2004). This central fatigue could originate from supra-spinal level or from inhibitory reflexes mediated by free endings of group III and IV afferents, stimulated by metabolites and damage to muscle spindles (Martin et al., 2005).

From a biomechanical perspective, foot-ground impacts cause sudden decelerations of soft-tissue packages inducing muscle oscillations. According to the “muscle tuning” paradigm, muscle activity is tuned in response to impact forces to dampen soft-tissue vibrations (Wakeling et al., 2001). During DHR, substantial increases in vertical impact force peaks (>50%) and horizontal braking force peaks (>70%) at steep slopes (i.e., –9°) were observed, compared to level running (Gottschall and Kram, 2005). Regarding the knee and ankle joints, which are considered as net absorbers and generators of force during DHR, the negative work period as a percentage of total stance time is significantly greater for these two joints during DHR than level running sessions (Eston et al., 1995). This longer negative work period combined with a reduced upward displacement of the center of mass causes a gradual disappearance of the bouncing mechanism during DHR as speed and slope become greater (Dewolf et al., 2016). Consequently, the negative work done by both KE and PF muscles is about twofold greater during DHR with a -8.3% slope than during level running as the same speed (4.5 m.s^-1^) (Buczek and Cavanagh, 1990). Therefore, KE or PF fatigue is greater after DHR than level running as a consequence of important absorption function and increased electromyographic activities (Giandolini et al., 2016a, 2017; Maeo et al., 2017). As a matter of fact, the increase in vertical downward velocity associated with higher ground reaction forces experienced during DHR might accentuate soft-tissue vibrations (Dewolf et al., 2016) and in turn, muscle activity. For instance, *triceps surae* soft tissue vibrations increased during prolonged and intense running sessions (Friesenbichler et al., 2011; Khassetarash et al., 2015). Since these findings were obtained during level treadmill running (∼40 min) at a relatively low velocity (from 3 to 4 m.s^-1^), one could assume that prolonged exposures of KE and PF to higher loading rate induced by a strenuous DHR may cause greater soft-tissue vibrations and in turn, increased electromyographic activity, which might contribute to greater EIMD and muscle fatigue.

On the physiological side, muscle fatigue may be associated with acute and delayed alterations in running economy (RE), i.e., oxygen demand for a given running speed, following trail running events (Vernillo et al., 2017) or laboratory-based DHR sessions (Chen et al., 2007). In a recent review, Vernillo et al. (2017) have suggested that muscle fatigue needs to be compensated by a greater neural input to the active muscles to produce the same amount of force, particularly during the push-off phase of the running step, leading to an altered RE (Vernillo et al., 2017). Interestingly, following a 65-km mountain ultramarathon, RE was significantly altered during downhill treadmill running whereas no significant changes in either level or uphill RE were observed. These results suggest that different contraction regimens specifically affect RE during exercise (Vernillo et al., 2015). It was also described that repeated and prolonged muscle eccentric actions induced by a 30-min treadmill DHR durably affect level RE at high metabolic intensities (>70%VO_2*max*_) in the recovery phase (up to 5 days after DHR) in untrained and moderately trained subjects (Chen et al., 2007, 2009).

Several strategies including DHR training sessions and the use of lower limb compression garments (CGs) have been tested in an attempt to reduce RE alterations and detrimental effects of muscle damage and/or muscle fatigue induced by trail running or DHR events (Bieuzen et al., 2014; Hill et al., 2014; Peake et al., 2017; Toyomura et al., 2017; Vercruyssen et al., 2017). Although recent reviews and meta-analyses indicated that wearing CGs during recovery may be effective in the attenuation of EIMD (Beliard et al., 2014; Brown et al., 2017; Hill et al., 2014), the beneficial effects of CGs on acute physiological responses during running are still debated.

In this regard, MacRae et al. (2011) reported that discrepancies in the findings might be population- and exercise-dependent (e.g., training status, treadmill slopes) or related to CGs features (e.g., intensity of compression). Using magnetic resonance imaging, Miyamoto and Kawakami (2014) found that wearing short tights with a high-pressure intensity of 15–20 mmHg reduced muscle fatigue during treadmill running. Additionally, a reduction in KE force decline was identified following a 15.6 km short trail running only for subjects wearing high-pressure compression stockings (>15 mmHg) during exercise (Bieuzen et al., 2014). The use of high-pressure CGs (>15 mmHg) might thus induce a beneficial effect on muscle damage during exercise and potentially, on RE. Investigations with participants not accustomed to DHR showed that the use of CGs may be an effective method to reduce muscle damage induced by DHR (Valle et al., 2013), by attenuating soft-tissue vibrations during exercise (Bieuzen et al., 2014). However, this mechanistic hypothesis has never been validated during eccentric endurance exercises either in recreational subjects or trained runners. The interest of wearing CGs in trail runners habituated to DHR, and for whom adaptations due to the repeated bout effects have already been induced by eccentric training (Hyldahl et al., 2017), remains to be elucidated. Therefore, it seems important to assess the effectiveness of wearing high-pressure CGs during DHR within a homogeneous group of well-trained trail runners habituated to eccentric contractions, through several outcome measurements including soft-tissue vibrations, RE, neuromuscular function, countermovement jump performance, and perceived muscle soreness.

Accordingly, the objective of the current work was to examine the effects of wearing high-pressure CGs (>15 mmHg) during a 40-min treadmill DHR on acute and delayed neuromuscular responses and RE in well-trained trail runners accustomed to eccentric work. We hypothesized that wearing CGs during exercise would reduce soft-tissue vibrations and thus, acute and delayed central and peripheral fatigue and improve RE.

## Materials and Methods

### Subjects

Thirteen well-trained male trail runners [(mean ± SD) age: 38.6 ± 5.7 years; height: 175.8 ± 5.1 cm; body mass: 72.1 ± 4.7 kg] participated to this study. Participants had a mean of 8.8 ± 3.4 years of trail running practice and were regularly involved in short-distance races (20–45 km). The average weekly training mileage during the two weeks before the first laboratory visit was 51.0 ± 20.6 km including 1913 ± 1181 m of positive/negative elevation. The sample size was calculated according to a previous study by Bieuzen et al. (2014) investigating the effect of a short trail running exercise [model inducing similar decrements in maximal voluntary contractions (MVC) than DHR] with the use of different running apparels on acute and delayed muscle fatigue (i.e., decline in MVC over time considered as the primary outcome), with a statistical power of 80% and a significance at *P* ≤ 0.05. Their mean VO_2*max*_ and maximal heart rate (HR_*max*_) were 64.6 ± 5.0 ml.kg^-1^.min^-1^ and 183.1 ± 8.1 beats.min^-1^, respectively. All subjects had previous experience with CGs for at least 2 years but none of them wore CGs on a regular basis during racing. This study was carried out in accordance with the recommendations of local institutional review committee (University of Toulon) with written informed consent from all subjects. All subjects gave written informed consent in accordance with the Declaration of Helsinki. The protocol was approved by the local institutional review committee (University of Toulon).

### Experimental Design

**FIGURE 1:**
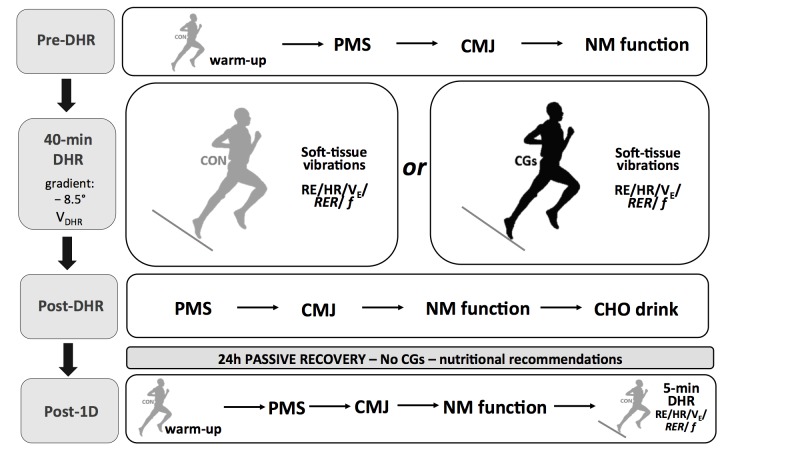
Experimental protocol. Schematic representation of the experimental design. Abbreviations: CON, control garments; CGs, compression garments; DHR, downhill running; *V_DHR_*, velocity associated with 55% VO_2*max*_; PMS, perceived muscle soreness; CMJ, countermovement jump; NM function, neuromuscular function; RE, running economy; HR, heart rate; *V_E_*, ventilation; RER, respiratory exchange ratio; *f*, step frequency.

Participants visited the laboratory on five different occasions. During the first visit, subjects performed a maximal test on a motorized treadmill (Venus 200/100r, HP cosmos, Germany) with a 10% slope that aimed at determining VO_2*max*_ and HR_*max*_. During this test, HR and breath-by-breath VO_2_ values were averaged every 10 s by the Oxycon Alpha metabolic measurement cart (Jaeger, Germany). VO_2*max*_ was determined from the three highest consecutive values (i.e., over a 30-s interval) reached during the last stage of the protocol. Following a 30-min recovery period which enabled to return to baseline VO_2_ and HR values (i.e., before the VO_2*max*_ protocol), subjects were instructed to run on the treadmill for the determination of velocity associated with 55% VO_2*max*_ of DHR (i.e., *V_DHR_*, –8.5°). In this session, subjects were familiarized with all experimental procedures. During the second and the fourth visits (separated by one week), athletes performed one 40-min treadmill DHR while wearing different running garments, with a set of measurements immediately before (Pre-) and after (Post-DHR). Each DHR session was followed by a similar set of measurements 1 day after (Post-1D, i.e., third and fifth visits) to evaluate the delayed effects of DHR (Figure 1). These blocks of 2 days (i.e., DHR + Post-1D) were performed in a counterbalanced and randomized order (using the following link: www.randomizer.org).

**FIGURE 2:**
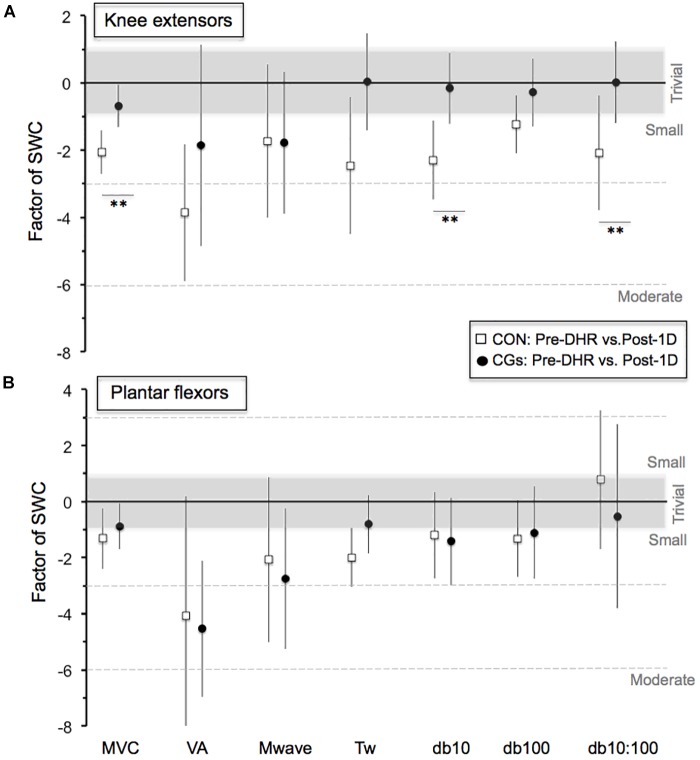
Magnitude of changes in the neuromuscular responses (Pre-DHR/Post-1D) for knee extensors **(A)** and plantar flexors **(B)** for control (CON) and compression garments (CGs) exercises. The standardized differences are expressed as a factor of the smallest worthwhile change [SWC = ES (Cohen’s *d*) of 0.2]. Bars indicate the 90% CLs. The number of asterisk (^∗^) indicates the likelihood for the between-exercise differences to be substantial, with ^∗^ indicating a *possible* and ^∗∗^ a *likely* difference. In the CGs exercise, the magnitude of changes in MVC, Db10, and Db10:100 for knee extensors was 1.5, 2.2, and 2.1 times greater than the SWC, respectively. Abbreviations: MVC, maximal voluntary contraction; VA, maximal voluntary activation; Mwave, peak-to-peak amplitude; Tw, potentiated twitch torque; Db10, low-frequency doublet force; Db100, high-frequency doublet force and Db10:100, low-to-high doublet frequency ratio.

Before starting DHR or Post-1D, subjects first carried out a warm-up which consisted of 7 min of level running (3.05 m.s^-1^) and 3 min of DHR (3.33 m.s^-1^; –10% slope). Then, perceived muscle soreness, countermovement jump, and neuromuscular function were evaluated in this order. During this set of measurements, subjects were asked to wear control garments (CON condition, loose-fitting conventional running garments, compression intensity <5 mmHg), whatever the running apparel assigned to DHR. After the neuromuscular protocol, subjects kept CON garments or wore lower limb CGs (CGs condition, SALOMON S/LAB EXO garments, stocking with 20–25 mmHg at the middle of calf and 18–20 mmHg at the upper site of calf, short-thigh with 16–18 mmHg at the middle of thigh, and 18–20 mmHg at the lower site of thigh) to begin DHR sessions. No “*in vivo*” CGs measurements (i.e., using a pressure sensor) were performed in our subjects during the DHR sessions. Prior to laboratory running sessions, CGs were re-fitted to obtain the required range of compression level according to manufacturer’s guidelines and based on subject’s circumference (i.e., upper, middle, and lower sites of thigh; middle and upper sites of calf) and limb lengths (Vercruyssen et al., 2017).

Running economy, HR, ventilation (*V_E_*), respiratory exchange ratio (RER), and step frequency (*f*) were determined at different time periods of DHR conditions but also, at *V_DHR_* (–8.5°) during the Post-1D run bouts. Accelerations of soft-tissue packages were exclusively measured during DHR conditions, allowing to assess soft-tissue vibrations at different time periods. At the end of each DHR, subjects were asked to report perceived muscle soreness scores and took off their CGs (for subjects wearing them during DHR) to complete a pair of countermovement jumps. No CGs were used during the set of measurements after DHR and within the recovery phase. The order of measurements in Post-1D was standardized as follows: perceived muscle soreness, countermovement jumps, neuromuscular function, and RE during the 5-min running bout at *V_DHR_* (Figure 1). Between the end of each DHR and the beginning of Post-1D testing bouts, participants were instructed not to perform any interventions including massage, icing and nutritional strategies (e.g., protein intake) possibly affecting the recovery process. Each subject received an isotonic carbohydrate (CHO)-sports drink (600 ml) after DHR and the quantity of CHO feedings was standardized (i.e., 8–10 g CHO per kg body mass) during the recovery phase. The training program was also standardized during the 7-day washout period separating the two exercise blocks (i.e., a 1-day passive rest between the end of DHR and Post-1D sessions for a given block but also, before the second DHR block, with intermediate sessions of 60-min swimming and 40-min low-intensity flat running at a mean HR <75% HR_*max*_). All experimental sessions were performed at the same time of day for a given subject and conducted between 10:00 and 16:00 hours under similar laboratory conditions (18–20°C, 35–40% relative humidity).

### Downhill Running

Following 3 min of high-intensity level running (i.e., corresponding to 3.88 m.s^-1^), the treadmill slope was immediately set to a –8.5° and *V_DHR_* was also set to induce the equivalent of a metabolic intensity of 55% VO_2*max*_. Then, the treadmill velocity was not changed within and between DHR exercises. Based on various pilot testing, *V_DHR_* was considered as a severe intensity which could be mechanically tolerated (in terms of repeated braking forces) by subjects during a 40-min period. According to Chen et al. (2009) and the reality of trail running, the gradient was set at –8.5° to induce substantial mechanical impairments. Mean *V_DHR_* represented 4.20 ± 0.23 m.s^-1^ during the DHR conditions and the Post-1D run bouts (–8.5°). During the DHR conditions, mean VO_2_ were 35.7 ± 3.5 ml.min^-1^.kg^-1^ for CON (i.e., 54.3 ± 4.6% VO_2*max*_) and 36.4 ± 2.0 ml.min^-1^.kg^-1^ for CGs (i.e., 55.5 ± 2.9% VO_2*max*_).

### Neuromuscular Function

The neuromuscular function was tested using the method of electrical stimulation as recommended by Millet et al. (2011a) similarly on the right KE and PF muscles in Pre, Post-DHR (7 min and 10-min after exercise termination for KF and/or PF), and Post-1D (same order of measurements than in Post for KF and PF). The evaluation of neuromuscular function was randomized for KE and PF in Post-DHR for a given condition. Before each test, the optimal stimulation intensity was identified by delivering successive single electrical stimuli at increasing intensities on relaxed muscles on the femoral (for KE) and tibial (for PF) nerves. The stimulation intensity used during all tests was 130% of optimal intensity to ascertain full spatial recruitment. The optimal stimulation intensities ranged from 110 to 160 mA for KE and from 110 to 130 mA for PF through the Pre-, Post-DHR, and Post-1D sessions. For the KE testing, subjects seated upright in a custom-built chair with hips at 100° of flexion and knees at 90°. The subjects’ ankle was strapped by non-compliant straps to the calibrated force transducer (F 501 TC 200 daN, TME 78 Orgeval, France) located slightly above the malleoli. The subjects were firmly attached to the ergometer with a rally car harness to avoid lateral and frontal displacements and were instructed to grip the harness during the test to standardize arm placement. For PF testing, subjects seated in the same custom-built chair and placed their foot, in a 0° dorsiflexion position, on a customized ergometer equipped with an instrumented pedal (SMTR 500 Nm, Sensel Measurement, Vincennes, France) located in the chair alignment. The forefoot was strapped to the pedal to limit heel lift and subjects were asked to perform a plantar flexion while keeping arms on their chest. The strain gauge and the pedal force were used to record the mechanical responses during MVC and electrically evoked contractions.

During the neuromuscular tests, transcutaneous electrical stimulations were applied to the femoral and posterior tibial nerves *via* a self-adhesive electrode cathode (10 mm in diameter) pressed manually by a researcher into either the femoral triangle (for KE) or the popliteal fossa (for PF) (Jubeau et al., 2017). The self-adhesive rectangular anode (50 mm × 90 mm, Dura-Stick Premium, Compex) was located either in the gluteal fold (for KE), or on the patella (for PF). A constant current stimulator (model DS7A, Digitimer, Hertfordshire, United Kingdom) delivered a square wave stimulus of 1 ms duration and 400 V maximal voltage and the interval of stimuli in the doublet were 100 and 10 ms for doublets at 10 Hz (Db_10_) and 100 Hz (Db_100_), respectively. Surface EMG signals were continuously recorded from the *vastus lateralis* and the *gastrocnemius medialis* muscles with a pair of self-adhesive surface (10 mm diameter) electrodes (Controle Graphique Medical, Brie-Comte-Robert, France) in bipolar configuration with a 20-mm interelectrode distance. The reference electrode was attached on the patella. Signals were amplified with a bandwidth frequency ranging from 1 Hz to 5 kHz (common mode rejection ratio = 110 dB, impedance input = 1000 MΩ, gain = 1000), digitized online at a sampling rate of 2000 Hz and stored for analysis with commercially available software (Acqknowledge 4.1, Biopac Systems Inc.).

For each condition, neuromuscular evaluation was conducted twice in Pre-, Post-DHR, and Post-1D. After a specific KE or PF isometric warm-up in both Pre- and Post-1D (i.e., 3-min submaximal contractions performed at increasing force levels), participants performed a similar neuromuscular evaluation for KE and PF which first consisted of a 4-s MVC followed by two single potentiated twitches separated by 2 s on the relaxed muscles. This procedure was repeated a second time after 15 s of rest. Following a resting period of 30 s, the subjects performed a third 4-s MVC superimposed with Db100 and followed after 2 s by two potentiated doublets in the relaxed muscle, i.e., Db100 and Db10, delivered 2 s apart. After 15 s of rest, this procedure was repeated a second time. The amplitude of the potentiated Db10, Db100, and the amplitude of the potentiated twitch peak torque (*T_W_*) that followed the two doublets as well as the ratio of paired stimulation peak forces at 10 Hz over 100 Hz (Db10:100) were analyzed for both KE and PF. Throughout the testing sessions, subjects were strongly encouraged during their MVC. On the contrary, they were asked to be as relaxed as possible during the peripheral fatigue measurements. For each variable, values were then averaged from the two series in Pre-, Post-DHR, and Post-1D.

The variability in VA was determined to assess central fatigue for KE and PF using a high-frequency doublet (100 Hz) superimposed on MVC. VA was calculated from the maximal force (*F_max_*) attained during the MVC, the force just before the superimposed doublet (*F_before_*), the peak force following the superimposed doublet (Db100*_sup_*), and control Db100 on relaxed muscle (Giandolini et al., 2016a) as follows:

(1) $$\text{VA}=[1-\frac{(\text{Db}100_{\sup}-F_{\text{before}})\times \frac{F_{\text{before}}}{F_{\max}}}{\text{control Db}100}]\times 100.$$

### Running Economy and Cardiorespiratory Parameters

Breath-by-breath VO_2_ values, *V_E_*, and RER were averaged every 10 s by the metabolic cart during overall DHR and Post-1D run sessions. For analysis, RE (expressed as VO_2_ for a given running velocity, in mlO_2_.min^-1^.kg^-1^), *V_E_*, and RER were averaged values from two time periods at the beginning (3–5 and 8–10 min) and the end of DHR (33–35 and 38–40 min) but also, during the 5-min running bout at *V_DHR_* (3–5 min) in Post 1-D. Using the HR sensor of the metabolic cart, HR values were also determined during the same time periods.

### Running Mechanics

Stride frequency was determined during the same time periods than for the RE measurements during DHR and in Post-1D (see above) using the app *Runmatic* recently validated (Balsalobre-Fernández et al., 2016) and installed on an iPhone 6 running iOS 11.0.3 (240 Hz high-speed camera, Apple Inc., Cupertino, CA, United States). To record the step periods, one operator lay prone on the ground, metricconverterProductID30?cm30?cm30 cm from the back of the treadmill (e.g., to analyze the back of the subjects’ feet), and held the iPhone in a vertical position at the same level as the floor of the treadmill. Then, contact time (*t_c_*) was calculated as the time between the first frame in which the foot contacts the treadmill and the first frame in which the foot takes off. Aerial time (*t_a_*) was calculated as the time between the first frame in which the foot takes off from treadmill and the first frame in which the other foot makes contact with the treadmill. Finally, *t_c_* and *t_a_* (in s) were averaged throughout eight consecutive steps (i.e., four stride cycles) and used to calculate step frequency (*f*, in Hz). The standard errors of estimate of the app *Runmatic*, compared to an opto-electronic device (*Optojump Next*) were 0.0056 s for contact time and 0.0048 s for aerial time.

### Soft-Tissue Vibrations

The current accelerometry method for quantifying soft-tissue vibrations has been previously validated in the context of running (Coza et al., 2010). Two lightweight tri-axial accelerometers (range = ± 200 g, mass = ± 5 g, TSD109, Biopac Systems, Inc., Goleta, CA, United States) were placed on the skin under the CON garments and CGs at the muscle belly of the *vastus lateralis* and *gastrocnemius medialis* of the right leg to quantify soft-tissue vibrations. Accelerometers were placed under CGs to maintain the permanent contact between the sites of skin and accelerometers. Accelerometers were attached using a double-sided adhesive and slightly plated with an adhesive tape to improve congruence with soft tissues without altering their motion. Their location was marked with indelible ink on the skin to enable reproducible accelerometer placement within-and between conditions. Acceleration signals were sampled at 1000 Hz, recorded for a 30-s interval at different time intervals of DHR and subsequently analyzed in Scilab 5.5.2 software (Scilab Enterprises, Orsay, France). To quantify the amount of soft-tissue vibrations, a time domain analysis was performed over the overall signals including both the stance and flight phases. The resultant acceleration (*A_r_*) was calculated from the three acceleration components for each muscle. The root mean square of the *A_r_* values (i.e., RMS *A_r_*) was then calculated and averaged using a 10-ms time window. For analysis, RMS *A_r_* for each muscle was finally averaged from two time periods at the beginning (4–5 and 9–10 min) and the end of DHR (34–35 and 39–40 min) to characterize the time effect on RMS *A_r_* within-and-between DHR sessions.

### Perceived Muscle Soreness

According to previous studies (Chen et al., 2009), perceived muscle soreness scores were assessed in Pre, Post-DHR, and Post-1D sessions, using a visual analogue scale consisting of a 100-mm continuous line anchored by “no pain” (metricconverterProductID0?mm0?mm0 mm) and “very, very painful” (metricconverterProductID100?mm100?mm100 mm). Subjects were asked to report the severity of global muscle soreness concerning the quadriceps and calves immediately after performing a five repetition sit–stand motion on each occasion.

### Countermovement Jump Performance

All participants were familiarized with countermovement jump testing during the first visit and completed countermovement jumps with hands on their hips, starting from a static position. Then, subjects performed a countermovement downward immediately followed by a complete extension of the lower limbs. During the flight phase of the jump, participants were instructed to jump as high as possible, and take-off and land with the feet simultaneously contacting the ground with the ankle in full dorsiflexion. The countermovement jump height was calculated using the app *My Jump* recently developed and validated by (Balsalobre-fernández et al., 2015). This app was installed on the same iPhone 6 used for the running mechanics recordings. To monitor the countermovement jump with *My Jump*, the device was installed onto the ground facing the subject’s feet (at ∼1.5 m). Take-off and landing frames of the video were used by the app for the calculation of the flight time and in turn countermovement jump height. Each athlete performed two countermovement jumps, separated by a 1-min passive rest period, in Pre (after a 10-min run warm-up), Post-DHR, and Post-1D sessions. The two countermovement jumps values were averaged for further statistical analysis. When compared to a force platform, Balsalobre-fernández et al. (2015) showed that a good reliability of the app for jump heights performed by different subjects (observer 1: α = 0.997, CV = 3.4%; observer 2: α = 0.988, CV = 3.6%). Furthermore, the Pearson product moment correlation coefficient showed almost perfect correlation between the app and the force platform measurements for jump height (*r* = 0.995, *P* < 0.001).

### Statistical Analysis

All dependent variables among CON and CGs exercises over the DHR/Post-1D blocks were initially tested for the normality of distribution and the homogeneity of variances using Shapiro–Wilk and Levene tests. Separate two-way ANOVAs (condition [CON, CGs] × time [Pre, Post, or Post-1D] and time periods over DHR) with repeated measures were applied to all dependent variables. This allowed quantification of the acute (Pre-Post) and delayed (Pre-Post 1-D) fatigue induced by DHR on dependent variables. When significant main effects were observed, Bonferroni’s test was used for *post-hoc* analysis. Within- and between exercise differences were also standardized from the use of Cohen’s effect sizes (ES) and thresholds [ 0.2 (*small*), >0.6 (*moderate*), >1.2 (*large*), and >2.0 (*very large*)] associated with 90% confidence limits (CL) to compare the magnitude of the difference of the change between pre- and post-DHR (immediate and post-1D) (Bieuzen et al., 2014; Hopkins et al., 2009). Probabilities that differences were higher, lower or similar to the smallest worthwhile difference (ES of 0.20) were evaluated qualitatively as follows: *possibly*, 25–74.9%; *likely*, 75–94.9%, *very likely*, 95–99.5%; *most (extremely) likely*, >99.5%. The true difference was assessed as *unclear* if the chance of both higher and lower values was >5% (Buchheit, 2016; Hopkins et al., 2009). The null hypothesis significance testing (NHST) was the primary method to discuss the current results and the qualitative approach (e.g., Cohen’s ES and smallest worthwhile changes) was used to further illustrate the differences between CON and CGs conditions, particularly when one clothing modality induced a significant effect over time. For all tests, an alpha of *P* < 0.05 was considered statistically significant. All qualitative analyses were conducted using modified statistical Excel spreadsheets (*via* www.sportsci.org).

## Results

All participants successfully completed the two blocks of DHR-Post-1D sessions, excepted for one subject where PF MVC values could not be analyzed for technical reasons. All standardized effects are presented as ES ± 90% CL. In order to ensure the CGs or CON conditions were performed in a comparable physical state, MVC for KE and PF but also, body mass were selected as standardization variables across exercises (in Pre-DHR). No significant differences were observed between conditions for the MVC responses for KE (*P* = 0.08) and PF (*P* = 1.00) or body mass (*P* = 1.00).

### Neuromuscular Function

**FIGURE 3:**
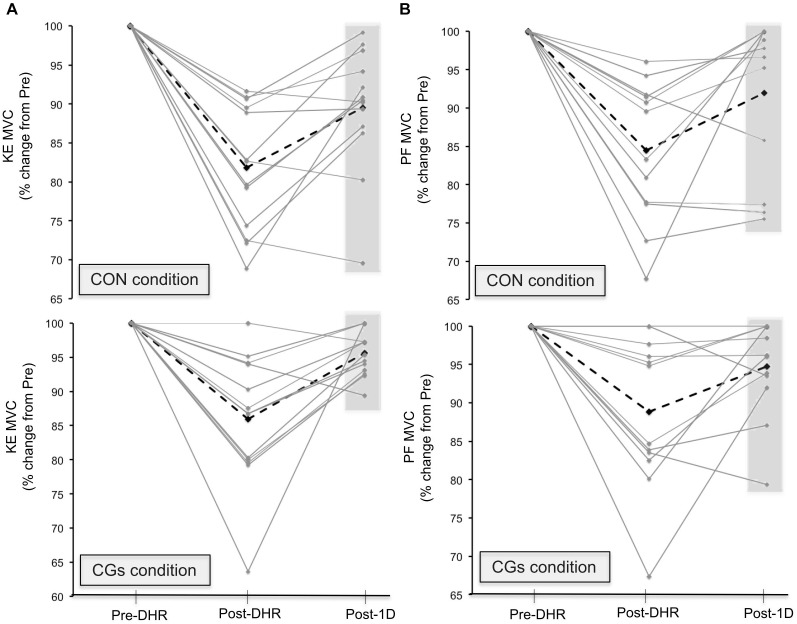
Individual responses in maximal voluntary contraction (MVC) torques for knee extensors [KE **(A)**] and plantar flexors [PF **(B)**] muscles in Pre-downhill running (DHR), immediate Post-DHR, and following 1 day recovery (Post-1D). Values are expressed in percentage change from Pre-DHR. The black dotted line indicates the average MVC value for a given condition. The gray area indicates subjects that may be “responders” or “non-responders” to CGs exercises in Post-1D sessions. A great percentage of positive responders to KE MVC recovery was identified in the CGs exercise. Abbreviations: CON, control garments; CGs: compression garments.

The changes in neuromuscular responses within-and-between exercise blocks are presented in Table 1 (i.e., Pre/Post-DHR) and Table 2 (i.e., Pre-DHR/Post-1D). Small to very large alterations (i.e., significant decreases) in acute neuromuscular responses, excepted for VL and GAST M-waves but also, for PF Db10:100, were found for both CON and CGs conditions, whatever the studied muscle (Table 1). A significant time × condition interaction was identified immediately after DHR for the KE VA deficit (*P* = 0.022). The KE VA deficit was significantly higher after CON than CGs, with a “moderate” standardized difference (ES = 0.83 ± 0.76, Table 1). The PF VA deficit significantly increased in the CON condition (*P* = 0.022), whereas no significant change in this variable was observed in the CGs condition (*P* = 0.398). Substantial delayed effects were also identified on neuromuscular responses for KE after CON condition while minor delayed effects were reported following CGs condition (Table 2). A significant time × condition interaction was found for decrements in KE MVC (*P* = 0.033), KE Db10 (*P* = 0.035), and KE Db10:100 (*P* = 0.042) which were significantly lower at 24-h after CGs condition, compared to CON condition. These differences in delayed neuromuscular responses were not reported for PF, excepted for *T_w_* values that were significantly lower following CON condition (*P* = 0.036). Given that the most beneficial effects of CGs were observed in Post-1D, the magnitude of Pre-DHR/Post-1D changes for all neuromuscular variables is presented in Figure metricconverterProductID2. In2. In addition, individual MVC responses for KE and PF across the two exercise blocks are displayed in Figure 3.

**Table 1:** Magnitude of Post-Pre downhill running (DHR) changes in neuromuscular variables for knee extensors and plantar flexors within- and between control garments (CON) and compression garments (CGs) conditions.

Downhill running (A Post-Pre)	CON condition							CGs condition					Post-Pre condition change		
	Pre-DHR	Post-DHR	Post-Pre DHR				Pre-DHR	Post DHR		Post-Pre DHR				CGs–CON conditions	

	Mean ± SD	Mean ± SD	%Δ ± SD ES ± 90% CL MBI	ES ± 90% CL MBI NHST (time x condition)	MBI	NHST	Mean ± SD	Mean ± SD	%Δ ± SD ES ± 90% CL MBI	ES ± 90% CL MBI NHST (time x condition)	MBI	NHST	ES ± 90% CL MBI NHST (time x condition)	MBI	NHST (time × condition)
Knee extensors															
MVC (Nm)	222.9	183.6	-18.2	-0.76	Moderate^∗∗∗∗^	*P* < 0.001	213.7	184.9	-13.9	-0.55	Small^∗∗∗∗^	*P* < 0.001	0.21	Small^∗^	*P* = 0.163
	±48.5	±48.3	±8.1	±0.16			±48.7	±49.3	±9.8	±0.19			±0.25		
VA (%)	93.8	86.5	-7.9	-1.37	**Large^∗∗∗∗^**	***P =* 0.001**	92.4	90.0	-2.4	-0.34	Unclear	*P* = 0.527	0.83	**Moderate^∗∗^**	***P* = 0.022**
	±4.9	±7.7	±5.1	±0.49			±6.5	±8.2	±8.4	±0.55			±0.76		
VL M-wave (mV)	8.2	6.9	-9.4	-0.67	Moderate^∗∗^	*P* = 0.743	7.9	7.2	-6.6	-0.37	Small^∗∗^	*P* = 1.000	0.22	Unclear	*P* = 0.692
	±1.3	±1.8	±18.9	±0.73			±1.6	±1.4	±14.7	±0.43			±0.82		
Tw (Nm)	48.9	31.2	-36.3	-3.20	Very large^∗∗∗∗^	*P* < 0.001	46.6	28.8	-38.0	-2.77	Very large^∗∗∗∗^	*P* < 0.001	0.00	Unclear	*P* = 0.996
	±5.2	±5.4	±9.0	±0.44			±6.0	±4.4	±6.9	±0.32			±0.53		
Db10 (Nm)	73.6	41.3	-43.7	-2.57	Very large^∗∗∗∗^	*P* < 0.001	70.2	38.3	-45.1	-2.47	Very large^∗∗∗∗^	*P* < 0.001	0.03	Unclear	*P* = 0.843
	±11.8	±9.2	±11.2	±0.40			±12.1	±7.4	±7.8	±0.34			±0.53		
Db100 (Nm)	75.6	62.3	-17.3	-1.10	Large^∗∗∗∗^	*P* < 0.001	72.7	60.2	-16.9	-0.97	Large^∗∗∗∗^	*P* < 0.001	0.07	Unclear	*P* = 0.723
	±11.3	±9.4	±8.2	±0.29			±12.0	±10.2	±7.3	±0.23			±0.37		
Db10:100 (%)	97.8	65.9	-32.0	-2.83	Very large^∗∗∗∗^	*P* < 0.001	97.1	63.7	-33.7	-2.63	Very large^∗∗∗∗^	*P* < 0.001	0.07	Unclear	*P* = 0.673
	±10.5	±8.8	±11.3	±0.57			±11.9	±6.7	±9.1	±0.47			±0.37		
Plantar flexors															
MVC (Nm)	132.1	111.5	-15.5	-0.63	Moderate^∗∗∗∗^	*P* < 0.001	137.0	121.6	-10.6	-0.41	Small^∗∗^	*P* = 0.001	0.16	Trivial^∗^	*P* = 0.225
	±30.5	±29.0	±9.2	±0.21			±34.8	±31.2	±10.8	±0.22			±0.31		
VA (%)	97.4	90.5	-7.0	-1.87	Large^∗∗∗^	*P* = 0.022	98.3	94.5	-3.9	-0.84	Moderate^∗∗∗^	*P* = 0.398	0.78	Unclear	*P* = 0.274
	±3.4	±8.5	±8.2	±1.16			±4.2	±5.5	±3.7	±0.42			±1.16		
GAST M-wave (mV)	10.4	9.5	-9.2	-0.53	Moderate^∗∗^	*P* = 0.477	10.6	9.5	-9.2	-0.54	Moderate^∗∗^	*P* = 0.245	-0.10	Unclear	*P* = 0.791
	±1.6	±2.7	±23.9	±0.64			±1.9	±1.7	±15.7	±0.37			±0.72		
Tw (Nm)	18.8	15.7	-15.6	-0.62	Moderate^∗∗∗∗^	*P* < 0.001	17.0	14.9	-10.7	-0.38	Small^∗∗^	*P* = 0.004	0.20	Small^∗^	*P* = 0.152
	±4.5	±3.8	±10.6	±0.24			±4.5	±4.1	±14.2	±0.24			±0.34		
Db10 (Nm)	33.9	27.7	-16.3	-0.47	Small^∗∗∗^	*P* = 0.005	32.5	25.0	-18.8	-0.52	Small^∗∗^	*P* = 0.001	-0.10	Unclear	*P* = 0.513
	±12.3	±10.0	±14.8	±0.23			±13.5	±10.4	±24.3	±0.34			±0.72		
Db100 (Nm)	36.8	33.0	-7.6	-0.26	Small^∗^	*P* = 0.037	35.3	29.8	-11.9	-0.35	Small^∗∗^	*P* = 0.003	-0.11	Unclear	*P* = 0.348
	±13.6	±11.1	±17.6	±0.26			±14.6	±12.0	±23.9	±0.30			±0.41		
Db10:100 (%)	92.3	84.0	-8.0	-0.89	Moderate^∗∗^	*P* = 0.270	92.9	83.9	-8.3	-0.62	Moderate^∗∗∗^	*P* = 0.191	-0.06	Unclear	*P* = 0.892
	±8.6	±10.7	±15.8	±0.83			±13.4	±7.0	±12.7	±0.40			±0.81		

*Values are expressed in percent change (%?) ± standard deviation (SD) and standardized effect size (ES) ± 90% confidence limits (CL). NHST, method of null-hypothesis significance testing (P < 0.05); MBI, method of magnitude based inferences; qualitative inferences are trivial (<0.20), small (0.20 to <0.60), moderate (0.60 to <1.20), large (1.20 to <2.00) and very large (>2.00): ^∗^possibly, 25 to <75%; ^∗∗^likely, 75 to <95%;^∗∗∗^very likely, 95 to <99.5%, ^∗∗∗∗^almost certain, >99.5%. MVC, maximal voluntary contraction; VA, maximal voluntary activation; Mwave, peak-to-peak amplitude; Tw, potentiated twitch torque; Db10, low-frequency doublet force; Db100, high-frequency doublet force and Db10:100, low-to-high doublet frequency ratio.*

**Table 2:** Magnitude of Post-1D-Pre downhill running (DHR) changes in neuromuscular variables for knee extensors and plantar flexors within- and between control garments (CON) and compression garments (CGs) conditions.

Recovery phase (A Post-1D-Pre)	CON condition							CGs condition					Post-Pre condition change		
	Pre-DHR	Post-DHR	Post-Pre DHR				Pre-DHR	Post DHR		Post-Pre DHR				CGs–CON conditions	

	Mean ± SD	Mean ± SD	%Δ ± SD ES ± 90% CL MBI	ES ± 90% CL MBI NHST (time x condition)	MBI	NHST	Mean ± SD	Mean ± SD	%Δ ± SD ES ± 90% CL MBI	ES ± 90% CL MBI NHST (time x condition)	MBI	NHST	ES ± 90% CL MBI NHST (time x condition)	MBI	NHST (time × condition)
Knee extensors															
MVC (Nm)	222.9	201.5	-10.4	-0.41	**Small^∗∗∗^**	***P* = 0.002**	213.7	206.6	-2.6	-0.14	Trivial^∗∗^	*P =* 0.707	0.29	**Small^∗∗^**	***P* = 0.033**
	±48.5	±54.5	±7.9	±0.13			±48.7	±43.3	±7.22	±0.13			±0.18		
VA (%)	93.8	89.7	-4.4	-0.77	Moderate^∗∗^	*P* = 0.463	92.4	89.8	-2.6	-0.37	Unclear	*P* = 1.000	0.29	Unclear	*P* = 0.622
	±4.9	±6.1	±4.2	±0.41			±6.5	±7.5	±8.8	±0.60			±0.73		
VL M-wave (mV)	8.2	7.7	-6.2	-0.35	Small^∗^	*P* = 1.000	7.9	7.3	-5.8	-0.36	Small^∗^	*P* = 1.000	0.07	Unclear	*P* = 0.836
	±1.3	±1.8	±14.0	±0.46			±1.6	±1.2	±16.4	±0.42			±0.63		
Tw (Nm)	48.9	46.2	-5.7	-0.49	Small^∗∗^	*P* = 0.238	46.6	46.6	0.2	0.01	Unclear	*P* = 0.703	0.48	Small^∗∗^	*P* = 0.123
	±5.2	±7.2	±9.6	±0.41			±6.0	±6.7	±8.3	±0.29			±0.48		
Db10 (Nm)	73.6	67.8	-8.5	-0.46	**Small^∗∗∗^**	***P* = 0.022**	70.2	69.7	-0.8	-0.03	Unclear	*P* = 1.000	0.44	**Small^∗∗^**	***P* = 0.035**
	±11.8	±15.0	±9.0	±0.23			±12.1	±13.2	±8.1	±0.21			±0.32		
Db100 (Nm)	75.6	72.6	-4.1	-0.25	Small^∗∗^	*P* = 0.175	72.7	72.0	-0.9	-0.06	Unclear	*P* = 1.000	0.19	Trivial^∗^	*P* = 0.208
	±11.3	±12.5	±5.9	±0.17			±12.0	±12.1	±7.3	±0.20			±0.27		
Db10:100 (%)	97.8	93.1	-4.6	-0.42	**Small^∗∗^**	***P* = 0.046**	97.1	97.2	0.2	0.00	Unclear	*P* = 1.000	0.42	**Small^∗∗^**	***P* = 0.042**
	±10.5	±11.0	±8.1	±0.34			±11.9	±11.8	±6.4	±0.24			±0.42		
PLANTAR FLEXORS															
MVC (Nm)	132.1	123.5	-7.0	-0.26	Small^∗^	*P* = 0.073	137.0	130.3	-4.1	-0.18	Trivial^∗^	*P* = 0.247	0.06	Unclear	*P* = 0.639
	±30.5	±35.0	±11.2	±0.22			±34.8	±29.3	±7.9	±0.16			±0.27		
VA (%)	97.4	94.4	-3.0	-0.81	Moderate^∗∗^	*P* = 0.319	98.3	94.3	-4.3	-0.91	Moderate^∗∗∗^	*P* = 0.076	-0.28	Unclear	*P* = 0.580
	±3.4	±5.7	±6.2	±0.86			±4.2	±8.2	±5.0	±0.49			±0.94		
GAST M-wave (mV)	10.4	9.7	-7.4	-0.42	Small^∗^	*P* = 1.000	10.6	9.5	-9.6	-0.55	Small^∗∗^	*P* = 0.449	-0.22	Unclear	*P* = 0.630
	±1.6	±2.7	±19.2	±0.59			±1.9	±2.3	±19.6	±0.50			±0.77		
Tw (Nm)	18.8	16.8	-11.3	-0.40	Small^∗∗^	*P* = 0.036	17.0	16.1	-3.5	-0.16	Trivial^∗^	*P* = 0.917	0.21	Small^∗^	*P* = 0.217
	±4.5	±5.3	±10.5	±0.21			±4.5	±4.3	±14.7	±0.21			±0.30		
Db10 (Nm)	33.9	30.7	-9.9	-0.24	Small^∗^	*P* = 0.899	32.5	28.4	-7.0	-0.28	Small^∗^	*P* = 0.414	-0.07	Unclear	*P* = 0.747
	±12.3	±15.3	±21.5	±0.31			±13.5	±10.1	±23.8	±0.31			±0.45		
Db100 (Nm)	36.8	33	-11.8	-0.26	Small^∗^	*P* = 0.539	35.3	31.8	-5.0	-0.22	Small^∗^	*P* = 0.737	0.03	Unclear	*P* = 0.896
	±13.6	±16.6	±18.9	±0.27			±14.6	±12.7	±25.5	±0.33			±0.44		
Db10:100 (%)	92.3	93.7	2.0	0.16	Unclear	*P* = 1.000	92.9	91.4	0.5	-0.10	Unclear	*P* = 1.000	-0.26	Unclear	*P* = 0.509
	±8.6	±9.0	±9.9	±0.50			±13.4	±12.4	±22.0	±0.66			±0.90		

*Values are expressed in percent change (%?) ± standard deviation (SD) and standardized effect size (ES) ± 90% confidence limits (CL). NHST, method of null-hypothesis significance testing (P < 0.05); MBI = method of magnitude based inferences; qualitative inferences are trivial (<0.20), small (0.20 to <0.60), moderate (0.60 to <1.20), large (1.20 to <2.00), and very large (>2.00): ^∗^possibly, 25 to <75%; ^∗∗^likely, 75 to <95%;^∗∗∗^very likely, 95 to <99.5%, ^∗∗∗∗^almost certain, >99.5%. Abbreviations: MVC, maximal voluntary contraction; VA, maximal voluntary activation; Mwave, peak-to-peak amplitude; T*_w_*, potentiated twitch torque; Db10, low-frequency doublet force; Db100, high-frequency doublet force; Db10:100, low-to-high doublet frequency ratio.*

### Soft-Tissue Vibrations

**FIGURE 4:**
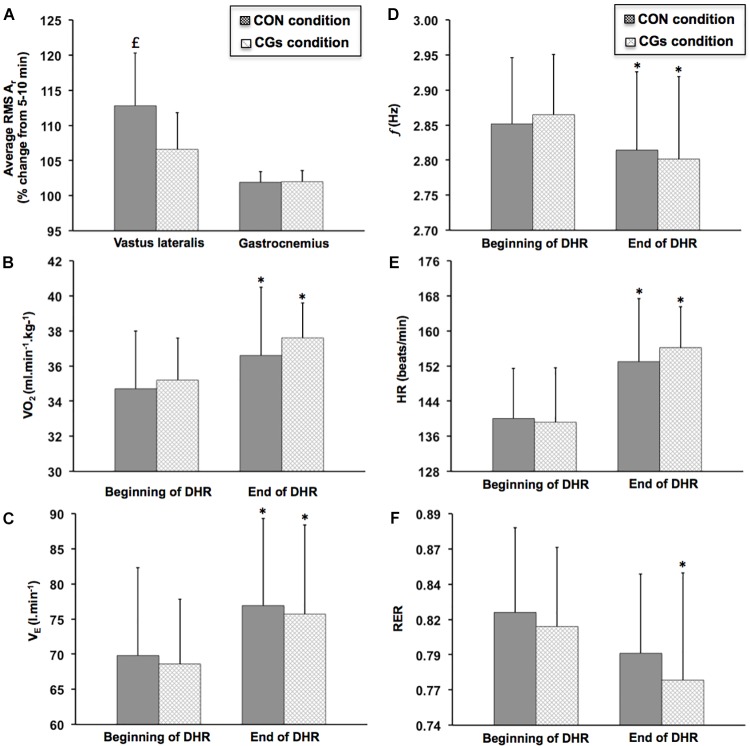
Changes in average resultant acceleration for *vastus lateralis* and *gastrocnemius medialis* [RMS *A_r_* **(A)**], oxygen uptake [VO_2_ **(*B*)**], ventilation [*V_E_* **(C)**], step frequency [*f* **(D)**], heart rate [HR **(E)**], and respiratory exchange ratio [RER **(F)**] during the 40-min downhill runs performed with control garments (CON) and compression garments (CGs). RMS *A_r_* was expressed as a function of time. VO_2_, *V_E_*, *f*, HR, and RER were averaged from two time periods at the beginning (3–5 and 8–10 min) and the end of DHR (33–35 and 38–40 min). ^∗^Significantly different than the beginning of DHR for a given condition (*P* < 0.05). significantly different than the CGs condition (*P* < 0.05).

Figure 4A shows changes in RMS *A_r_* over time for *vastus lateralis* and *gastrocnemius medialis* within CON and CGs conditions. A significant increase in RMS *A_r_* was observed over time for the *vastus lateralis* in the CON condition (+11.6 ± 5.9%; ES = 0.69 ± 0.24; *P* = 0.003), while no significant difference in this variable was observed over time in the CGs condition (+6.6 ± 5.2%; ES = 0.33 ± 0.16, *P* = 0.121). The increase in RMS *A_r_* was *likely* higher after CON than CGs conditions, with a “moderate” standardized difference between conditions (ES = –0.86 ± 0.71). No significant differences in RMS *A_r_* were found for the *gastrocnemius medialis* within-and-between exercises (*P* = 0.246).

### Running Economy, Cardiorespiratory Parameters, and Stride Frequency

The most significant changes in VO_2_, *V_E_*, HR, RER, and *f* were observed at the end of DHR in both CON and CGs conditions (Figure 4) while no significant differences in these variables were found in Post-1D, as compared to the beginning of each DHR (Table 3). At the end of DHR, a significant increase was observed in both conditions (i.e., time effect) for VO_2_ (CON: +5.3 ± 6.0%; ES = 0.51 ± 0.31, *P* = 0.049 and CGs: +6.8 ± 5.6%; ES = 0.91 ± 0.41; *P* = 0.012), *V_E_* (CON: +11.0 ± 11.3%; ES = 0.53 ± 0.27, *P* = 0.013 and CGs: +10.3 ± 9.6%; ES = 0.71 ± 0.36; *P* = 0.013), and HR (CON: +9.3 ± 5.5%; ES = 1.04 ± 0.35; *P* = 0.002 and CGs: +12.6 ± 7.4%; ES = 1.26 ± 0.39; *P* < 0.001). In addition, RER values were significantly lower over time only for CGs (–4.9 ± 4.4%; ES = –0.63 ± 0.31; *P* = 0.004), whereas no significant change in this variable was observed for CON (–3.1 ± 2.2%; ES = –0.40 ± 0.15; *P* = 0.066). Similarly, *f* values were significantly lower over time only for CGs (–2.2 ± 2.1%; ES = –0.69 ± 0.32; *P* = 0.003), whereas no significant change in this variable was observed for CON (–1.3 ± 2.7%; ES = –0.37 ± 0.39; *P* = 0.119).

**Table 3:** Table 3 | Changes in oxygen uptake (VO_2_), ventilation (V_E_), heart rate (HR), respiratory exchange ratio (RER) and step frequency (*f*) between the 3–5 min interval of downhill running (DHR) in the control garments (CON) or compression garments (CGs) conditions and the 3–5 min interval of the running bout in Post-1D following CON and CGs.

Recovery phase Δ Post-1D-DHR (3–5 min	CON condition							CGs condition					Post-1D-DHR condition change		
	DHR	Post-1D		Post-1D-DHR			DHR	Post-1D		Post-1D-DHR				CGs–CON conditions	
	
	Mean ± SD	Mean ± SD	%Δ ± SD	ES ± 90% CL	MBI	NHST	Mean ± SD	Mean ± SD	%Δ ± SD	ES ± 90% CL	MBI	NHST	ES ± 90% CL	MBI	NHST (time × condition)
V0_2_ (ml.min^-1^.kg^-1^)	34.5	35.4	2.8	0.23	Small^∗^	*P* = 1.000	35.3	36.1	2.3	0.27	Unclear	*P* = 1.000	-0.06	Unclear	*P* = 0.839
	±3.5	±3.1	±4.4	±0.22			±2.4	±2.7	±9.0	±0.63			±0.53		
*V_E_* (l.min^-1^)	69.8	67.9	-1.3	-0.14	Unclear	*P* = 1.000	68.6	68.8	-0.7	0.02	Unclear	*P* = 1.000	0.18	Unclear	*P* = 0.573
	±12.5	±7.9	±11.3	±0.35			±9.2	±10.1	±11.9	±0.39			±0.53		
HR (beats.min^-1^)	140.1	135.6	-3.2	-0.36	Small^∗∗^	*P* = 0.713	139.2	138.4	-1.2	-0.14	Unclear	*P* = 1.000	-0.29	Small^∗^	*P* = 0.525
	±11.5	±13.1	±4.5	±0.28			±12.3	±10.6	±6.4	±0.39			±0.35		
RER	0.82	0.78	-4.0	-0.60	Moderate^∗∗^	*P* = 0.454	0.81	0.80	-0.9	-0.16	Unclear	*P* = 1.000	0.48	Unclear	*P* = 0.328
	±0.06	±0.03	±8.6	±0.63			±0.06	±0.03	±0.03	±0.45			±0.79		
*f* (Hz)	2.86	2.91	1.9	0.52	Small^∗∗^	*P* = 0.120	2.86	2.88	0.7	0.22	Small^∗^	*P* = 1.000	-0.34	Unclear	*P* = 0.239
	±0.10	±0.13	±3.5	±0.49			±0.09	±0.10	±1.6	±0.25			±0.58		

*Values are expressed in percent change (%?) ± standard deviation (SD) and standardized effect size (ES) ± 90% confidence limits (CL). NHST, method of null-hypothesis significance testing (P < 0.05); MBI, method of magnitude based inferences; qualitative inferences are trivial (<0.20), small (0.20 to <0.60), moderate (0.60 to <1.20), large (1.20 to <2.00), and very large (>2.00): ^∗^possibly, 25 to <75%; ^∗∗^likely, 75 to <95%;^∗∗∗^very likely, 95 to <99.5%, ^∗∗∗∗^almost certain, >99.5%.*

### Perceived Muscle Soreness and Jump Performance

The magnitude of changes in perceived muscle soreness reported after CON and CGs conditions and in Post-1D is presented in Figure metricconverterProductID5. A5. A significant time × condition interaction was identified for scores in perceived muscle soreness only at quadriceps level (*P* = 0.026) which were significantly lower in Post-1D following CGs compared to CON, whereas no significant time (*P* = 0.069) or time × condition interaction effects (*P* = 0.117) were found at calves level. In addition, a significant decrease in countermovement jump was observed for CON (–6.7 ± 7.1%; ES = –0.57 ± 0.32; *P* = 0.003) and a strong trend for a decrease was identified for CGs (–4.2 ± 8.4%; ES = –0.31 ± 0.26; *P* = 0.073) following DHR. The significant alterations in jump performance were also identified in Post-1D for CON (–7.7 ± 5.8%; ES = –0.68 ± 0.26; *P* = 0.003) and CGs (–5.1 ± 7.5%; ES = –0.39 ± 0.24; *P* = 0.039) conditions. The mean values in countermovement jump were 36.2 ± 4.0 and 35.2 ± 4.9 cm in Pre-DHR, 33.2 ± 3.4 and 33.6 ± 4.3 cm in Post-DHR, 33.3 ± 3.0 and 33.2 ± 3.3 cm in Post-1D for CON and CGs conditions, respectively.

## Discussion

We hypothesized that wearing high-pressure CGs during an intense downhill run would attenuate soft-tissue vibrations, acute and delayed alterations in muscle function and improve RE. Our hypotheses have been partially confirmed, as the most important findings of this study are (i) an attenuation of soft-tissue vibrations during DHR (only for the *vastus lateralis*) and a reduced VA deficit (only for KE) in the CGs condition, (ii) a deterioration in RE for both CGs and CON conditions, and (iii) a faster recovery of MVC and peripheral parameters at 24 h post-CGs with lesser muscle soreness (only for KE).

### Acute Effects of Wearing Compression Garments

A reduction in MVC is a well-acknowledged and reliable index for assessing muscle damage within a whole muscle group (Damas, 2016). The magnitude of MVC decline appears to be directly related to the number of muscle fibers with myofibrillar disruption and/or excitation-contraction coupling failure (Peake et al., 2017; Raastad et al., 2010). *Small* to *moderate* decreases in MVC were observed immediately after DHR either in CGs or CON condition for KE (–13.9 ± 9.8 and –18.2 ± 8.1%, respectively) and PF muscles (–10.6 ± 10.8 and –15.5 ± 9.2%, respectively, Table 1). The significant decrements in MVC, whatever the condition, were lower than those measured in previous downhill studies using a 30-min treadmill exercise (∼ –20% for KE) (Chen et al., 2007) or a 6.5 km downhill trail run (-18.6% for KE and -25.4% for PF) (Giandolini et al., 2016a). The observed discrepancies between studies are likely due to the differences in DHR training status of the subjects. Indeed, the subjects recruited in the present study had a high trail running background and/or practice and hence, all were highly accustomed to DHR before the starting of laboratory sessions. Overall, the acute MVC responses to the high DHR intensity (∼4.2 m.s^-1^) might be specific to the training status of our population and thus provide valuable insights on the evaluation of muscle function following DHR in trained subjects.

Maximal voluntary contractions loss for KE and PF following DHR is usually related to alterations in both central and peripheral factors (Giandolini et al., 2016b). The current findings indicate small to large alterations in VA and peripheral variables (M-wave or Db10:100) for KE and PF, independently of the experimental condition, with greater changes in the peripheral components (Table 1). These data are consistent with those reported in previous laboratory and ecological downhill studies (Giandolini et al., 2016a; Martin et al., 2004). Thus, eccentric exercise such as DHR is known to induce severe lower limb tissue damage and low-frequency fatigue (i.e., decreased Db10:100), particularly for KE. Larger decreases in Db10:100 for KE (from –32.0 to –33.7%) compared to PF (from –8.0 to –8.3%) were observed in the present study, which are in line with those recently observed following a 6.5-km downhill trail run (Giandolini et al., 2016a). Several mechanisms are proposed to characterize peripheral fatigue, including impairments in sarcolemmal action potential conduction and excitability (Piitulainen et al., 2008, 2010), depressed Ca^2+^ release from the sarcoplasmic reticulum (Hill et al., 2001; Martin et al., 2005) but also decreased in Ca^2+^ sensitivity and/or force produced by active cross-bridges (Place et al., 2010). Although using CGs has been shown to reduce muscle activation during prolonged level running, which might improve muscle function (Hsu et al., 2017), results of the present work do not support a beneficial effect of CGs on peripheral fatigue for KE and PF. The delay time for measuring muscle function (7–10 min after exercise termination) constitutes a methodological limitation, which may have counteracted the potential benefits of CGs on the extent of peripheral fatigue. In this regard, Froyd et al. (2013) demonstrated a rapid recovery of peripheral variables within the first 8 min of the recovery period, thus underestimating not only the extent of peripheral fatigue in our study (and previous reports) but also the potential beneficial effect of CGs.

Surprisingly, the most noticeable acute effect of wearing CGs was observed on the VA deficit. Indeed, the KE VA deficit was significantly lower following the CGs condition, compared to the CON condition (–2.4 vs. –7.9%, respectively, Table 1). It is well documented that central drive is controlled by a combination of factors including excitatory and inhibitory reflex inputs from muscles, joints, tendons, and cutaneous afferents (Amann et al., 2015; Millet et al., 2012). Hence, an attenuated VA deficit, as reported following the CGs condition, could be attributed to a higher output from the motoneuron pool resulting not only from decreased inhibitory actions (group III/IV muscle afferents) but also through facilitation of Ia afferents inputs onto alpha motoneurons (Souron et al., 2017). In a recent literature review, Souron et al. (2017) reported that Ia afferents, which innervate muscle spindles, are the most responsive receptors to local vibratory stimuli, especially when muscles are stretched (as during DHR). Interestingly, a significant time effect on RMS *A_r_* values was identified, indicating an increase in RMS *A_r_* and thereby, soft-tissue vibrations over time for the *vastus lateralis* only in the CON condition (Figure 4A). Although no significant interaction effect was observed, the change in RMS *A_r_* was *likely* higher after CON than CGs conditions with a “moderate” standardized difference between conditions (ES = –0.86 ± 0.71). Wearing CGs at the quadriceps level may have exerted dynamic immobilization reducing muscle oscillation and improving joint stability, and in turn, enhancing neural input (Doan et al., 2003; Kraemer et al., 2001). Although the analysis was limited to the *vastus lateralis*, the decrease in soft-tissue vibrations only in the CGs condition, that reflects an attenuation of impact forces (Friesenbichler et al., 2011), might contribute to the reduced VA deficit in the CGs condition. While vibrations were not local but naturally extended to overall quadriceps during DHR, we suggest that the decrease in soft-tissue vibrations for the *vastus lateralis* only in the CGs condition influences the feedback from muscle spindle afferents and reduces central drive alteration. Future mechanistic approach is necessary to better understand the mechanisms underlying the relationship between soft-tissue vibrations, muscle fatigue and CGs.

To date, laboratory studies quantifying soft-tissue vibrations were limited to level treadmill running during which no evaluation of muscle fatigue was carried out (Friesenbichler et al., 2011; Khassetarash et al., 2015). During level running, it has been suggested that muscles actively participate to the shock and vibration attenuation, according to the paradigm of “muscle tuning” proposed by (Nigg and Wakeling, 2001). In this paradigm, pre-activation and muscle activation intensities are adapted in accordance with the impact magnitude at ground contact in order to control soft-tissue vibrations (Boyer and Nigg, 2004; Wakeling et al., 2001). However, higher values in peak vertical impact and loading rate were observed during DHR exercises compared to uphill or level running exercises (Gottschall and Kram, 2005; Mizrahi et al., 2000), with an accentuated involvement of “muscle tuning” in such conditions. In the current study, we suggest that the protective mechanism of “muscle tuning” is accentuated in the CGs condition, especially on muscle mass substantially involved in shocks absorption (e.g., KE muscles). Indeed, a significant variation in soft-tissue vibrations (e.g., RMS *A_r_*) was observed over time for the *vastus lateralis* only in the CON condition whereas no significant difference in RMS *A_r_* was found for the *gastrocnemius medialis* within-and-between exercises (Figure 4A). It has been previously reported a greater eccentric work (in relation with higher volume and/or mass) for KE compared to PF muscles during DHR (Buczek and Cavanagh, 1990), which might be associated with higher shock absorption, thus locally affecting the magnitude of soft-tissue vibrations. Interestingly, when focused on the *vastus lateralis*, the difference between DHR conditions was *likely* beneficial in favor of CGs, as shown by the lower increase in RMS *A_r_* over time (–5%) and the large ES (–0.86 ± 0.71) for this condition. Considering these results, wearing CGs during DHR might constitute a mechanical strategy to accentuate the muscle damping of soft-tissue vibrations especially for KE.

However, the beneficial effects of CGs on soft-tissue vibrations or VA were not associated with improved perceived muscle soreness scores and countermovement jump responses following DHR. Muscular power and strength as well as muscle soreness are the most common markers used to assess EIMD following eccentric exercises (Hill et al., 2014). It has been demonstrated that muscle soreness, reflecting connective tissue damage and inflammation in the extracellular matrix (Peake et al., 2017), appears to be independent of other markers such as MVC (Nosaka et al., 2002). In the present study, the lack of differences in perceived muscle soreness scores between sessions immediately after DHR might be explained by the high DHR intensity sustained for both conditions (thus affecting immediately perceived muscle soreness responses) and/or the short delay for measuring perceived muscle soreness after DHR (∼2 min). Indeed, perceived muscle soreness are known to increase in the hours following eccentric exercises and peak after 1–3 days (Cheung et al., 2003). Otherwise, although significant decreased (or strong tendency) countermovement jump height were found after DHR, no difference in jump performance was identified between sessions, confirming previous results obtained after simulated trail running races (Kerhervé et al., 2017; Vercruyssen et al., 2014). Considering these elements, MVC appears to be the most sensitive marker to assess acute effects of DHR on muscle damage (Damas, 2016).

Finally, altered RE, *V_E_*, RER, and HR responses were observed over time and regardless of conditions (Figure 4), suggesting a relationship between these cardiorespiratory parameters. As previously reported by Chen et al. (2007), it is likely that the changes in HR, *V_E_*, and RER are indicative of an altered RE (i.e., significant increase in the VO_2_ response) among DHR sessions. For instance, HR values were significantly higher over time (from +9.3 to +12.6%, Figure 4E) that might be related to the VO_2_ drift but also, to the potential increase of core body temperature experienced during DHR (Westerlind et al., 1992). Time course of RE responses during DHR are relatively scarce in literature (Dick and Cavanagh, 1987; Gavin et al., 2015; Westerlind et al., 1992), with no reports in trained runners accustomed to eccentric work. In the current investigation, we reported a 5.3–6.8% increase in RE (Figure 4B) at the end of DHR for both conditions. These RE alterations are lower than those reported in previous studies (Dick and Cavanagh, 1987; Westerlind et al., 1992) indicating substantial increases in RE (expressed as VO_2_) over time (>+10%), probably due to the low training status of the subjects. Moreover, stride pattern can affect RE during fatiguing and non-fatiguing running (Moore et al., 2016). For instance, we reported a significant decrease in *f* (Figure 4D) in the CGs condition that might be related to the significant increase in RE in this condition. These findings are in agreement with previous work showing a similar RE-stride pattern relationship in trained subjects running for 1 h at the fastest possible speed (Hunter and Smith, 2007). This suggests that subjects of the present work modify their RE responses while adjusting an optimal stride pattern with fatigue. In addition, several hypotheses may be proposed to explain the altered RE over time for both DHR exercises, including an increased motor unit recruitment to maintain prolonged DHR exercises and/or preferential type II fiber recruitment (Dick and Cavanagh, 1987; Douglas et al., 2017) but also, substantial normal impact force and parallel braking force peaks (Gottschall and Kram, 2005). Therefore, RE responses in the present study are consistent with recent investigations reporting changes in RE responses independently of wearing CGs either during level running of short duration or prolonged trail running in well-trained populations (Stickford et al., 2015; Vercruyssen et al., 2017). Based on a recent study focusing on the relationship between downhill training and chronic RE responses (Shaw et al., 2018), RE does not appear to be the most sensitive index to evaluate the efficacy of an intervention in already well-trained subjects.

### Delayed Effects of Wearing Compression Garments

This study is the first to demonstrate that the completion of a DHR exercise while wearing CGs may exert meaningful differences between acute and delayed neuromuscular responses. In contrast with acute effects of CGs described above, peripheral alterations were strongly reduced for KE compared to PF muscles at 24 h post-CGs (Table 2), suggesting that the use of CGs during strenuous DHR may constitute a strategy for runners to enhance subsequent recovery of KE muscles. All subjects wore CGs only during DHR, thus excluding the potential recovering effect of wearing CGs in the hours following eccentric exercises (Hill et al., 2014). Reductions in KE MVC were –2.6% at 24 h post-CGs (vs. –10.4% at 24 h post-CON), and were substantially lower that those reported at 24-h following 30–45 min DHR exercises (–17.0%) in physically active subjects (Maeo et al., 2017; Malm et al., 2004) or after a 6.5 km downhill trail run session (–8.5% at post-48 h) in trained runners (Giandolini et al., 2016a). These discrepancies in MVC responses between studies at 24 h post-DHR may be mainly explained by differences in training status. All participants in the present work had a high DHR training status and thus, a better recovery capacity for this exercise modality (Douglas et al., 2017; Hyldahl et al., 2017). For instance, the percentage of positive responders to MVC recovery was particularly elevated for KE (Figure 3) and might represent a specificity of our population.

This delayed effect of CGs on the reduction in MVC loss is consistent with those previously reported (Bieuzen et al., 2014), indicating *likely* and *possibly* beneficial effects of CGs on MVC losses at 1 and 24 h post-trail running, respectively. These authors have related the delayed benefits of CGs to the reduction in muscle oscillation and/or mechanical stress induced during trail running. In support of this hypothesis, the reduction in soft-tissue vibrations observed only in the CGs condition for the *vastus lateralis* may have contributed to the improvement of muscle recovery, notably by reducing peripheral alterations. Interestingly, a rapid recovery in Db10 and Db10:100 variables for KE was also observed at 24 h post-CGs (Table 2), suggesting a reduced failure in excitation–contraction coupling mechanisms. Such restoration of these neuromuscular variables was not observed at 24 h post-CGs for PF, excepted for *T_w_* values. From a mechanistic perspective, future research is warranted to evaluate, within the recovery phase, the vibration damping properties which may vary with fatigue (Friesenbichler et al., 2011; Khassetarash et al., 2015) and its relationship with EIMD.

**FIGURE 5:**
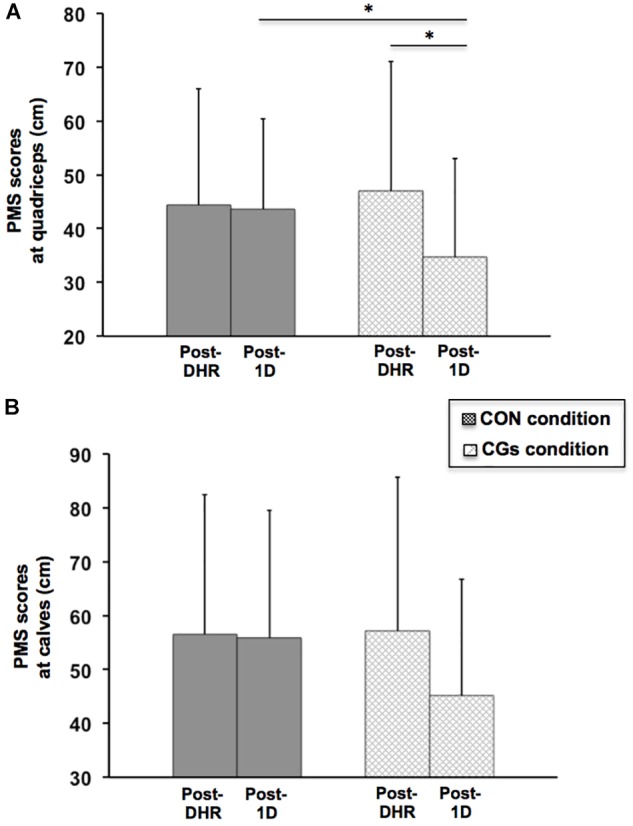
Perceived muscle soreness (PMS) scores at quadriceps **(A)** and calves **(B)** levels in the control garments (CON) and compression garments (CGs) conditions, after DHR, and during the recovery process (Post-1D). ^∗^Significantly different from Post-DHR for a given condition but also from the same period when compared to the CON condition (*P* < 0.05).

Previous theories have been proposed to describe the extent of EIMD, including sarcomeres disruption (e.g., the “popping sarcomere” hypothesis”) or damage to the excitation–contraction coupling system (Morgan and Proske, 2004; Proske and Morgan, 2001). In a recent review, Douglas et al. (2017) have reported that overstretched sarcomeres induce ultrastructural myofibrillar disruption, overloading sarcolemma and t-tubules structures and in turn, excitation–contraction coupling dysfunction. In addition, extensive EIMD may lead to inflammatory syndrome, triggering nociceptor stimulation (group III and IV afferents) and subsequently, muscle soreness (Peake et al., 2017; Proske and Morgan, 2001). Considering these statements, we assume that wearing CGs during an intense and prolonged DHR may have “mechanically” preserved KE muscles, which are strongly exposed to muscle damage and peripheral fatigue during DHR (Giandolini et al., 2016b; Maeo et al., 2017). This “protective effect” exerted by the use of high-pressure CGs might be effective especially in the hours following exercise where EIMD symptoms begin to spread intensively within skeletal muscles (Peake et al., 2017). This hypothesis is consistent with perceived muscle soreness scores reported for quadriceps (Figure 5), that were significantly lower at 24 h post-CGs, whereas no significant changes in perceived muscle soreness were found between conditions after DHR. While countermovement jump performances did not change within the recovery phase, perceived muscle soreness index seems to be more sensitive in the hours following DHR and might be related to lesser peripheral alterations. Based on reviews considering CGs as a strategy for enhancing recovery from muscle damage and inflammation (Brown et al., 2017; Dupuy et al., 2018; Peake et al., 2017), we suggest that wearing high-pressure CGs (especially for KE) during DHR contribute to the enhanced muscle recovery process by exerting an exercise-induced “mechanical protective effect”. Further research is warranted to know whether this “protective effect” may be potentiated by the use of CGs during the recovery phase, notably on KE muscles.

The reduction of EIMD and peripheral fatigue at 24 h post-CGs was not associated with responses in RE and cardiorespiratory parameters (Table 3) that were unchanged, whatever the condition. These findings are consistent with RE responses reported 24 h after the completion of a 30-min DHR exercise in physically active males (Mizuno et al., 2016). However, in this investigation, CGs were not used during DHR (only during the 24 h recovery period), making the comparison with our data difficult. Regardless of the use of CGs, it has been reported that DHR induced an alteration in RE at 48 h post-exercise in well-trained runners and triathletes (Braun and Dutto, 2003), which lasted for 3 days in soccer players (Chen et al., 2007) and 5 days in untrained subjects (Chen et al., 2009). In these studies, altered RE responses were associated with increases in EIMD markers (e.g., MVC, creatine kinase activity) or changes in stride mechanics (e.g., increased stride frequency), highlighting a relationship between RE and muscle damage in populations unaccustomed to DHR exercises. Although our experimental design was quite different than those used in these previous investigations, the present work indicates no significant differences in RE and cardiorespiratory responses in Post-1D among conditions, as compared to the first 5-min of DHR, and shows that during the recovery phase, these variables returned rapidly to values obtained at the beginning of DHR in highly trained runners. On the opposite, *f* values were *significantly* higher in Post-1D, as reported in previous work (Chen et al., 2007). Considering acute and delayed RE responses, we postulate that RE is a too “robust” indicator to be sensitive to certain external strategies, such as wearing CGs, in already well-trained subjects (Shaw et al., 2018).

Some methodological limitations must be considered such as the delay time for measuring muscle fatigue (7–10 min after DHR), possibly counteracting not only the potential benefits of CGs on acute central and peripheral adaptations (Froyd et al., 2013) but also the lack of mechanistic explanation of the “protective effect” observed in the CGs condition and its consequences on muscle damage and peripheral fatigue during the recovery phase. Within this framework, the temporal analysis of soft-tissue vibrations only during DHR and limited to the *vastus lateralis* as well as the lack of calculation of damping characteristics did not allow to infer on the potential relationship between vibrations and muscle function in the CGs condition and the recovery phase. It has been shown that soft tissue vibrations tend to increase as fatigue develops from a level running protocol (Friesenbichler et al., 2011). In this regard, a methodology based on the complex analysis of vibratory properties (Enders et al., 2012) might be useful to determine whether the musculoskeletal system is able to further dampen the increased vibration amplitude with the use of CGs, and how vibratory properties may act, in turn, on muscle damage and peripheral fatigue during and after exercise. Additionally, it would be interesting in future investigations to use histological analyses of muscle biopsies (Valle et al., 2013) or transverse relaxation time-weighted magnetic resonance imaging (Maeo et al., 2017) to better understand the impact of wearing CGs on the extent of muscle damage (e.g., sarcomeres disruption) and inflammatory edema following DHR. Finally, although a great majority of beneficial effects was reported on KE in the hours following DHR, it remains however uncertain whether such benefits are primarily linked to the use of thigh compression during DHR or if the “protective effect” results in the combined effects of calves and quadriceps compression. Using different apparel strategies, further work could isolate the responses of each muscular compartment compressed on muscle damage and/or muscle fatigue during running and within the recovery phase.

## Conclusion and Perspectives

This study shows that the use of high-pressure CGs during DHR induces beneficial effects on soft-tissue vibrations, acute and delayed neuromuscular responses, and muscle soreness, in well-trained off-road runners. The attenuation of soft-tissue vibrations only in the CGs condition might contribute, at least in part, to the reduced VA deficit immediately after DHR and, to the improved muscle function during the recovery phase. This study suggests that the use of CGs exerts an exercise-induced “mechanical protective effect,” that might constitute an external strategy for runners, especially to tolerate a high training load or to optimize recovery process within multi-stage races. Given that our findings were observed in highly trained trail runners, we assume that the observed effects with the use of CGs would be even greater in less trained or recreational subjects. Future studies are required to better understand the extent to which wearing CGs may alter the degree of muscle damage or reduce decrements in central and peripheral fatigue-related variables for KE muscles. Moreover, a detailed analysis of the contribution of wearing CGs during DHR to inflammatory mechanisms would be interesting, since they are involved in cellular signaling allowing adaptation to muscle regeneration following training (Peake et al., 2017). It would thus be necessary to ensure that a usual wearing of CGs during training does not interfere with muscular adaptation, especially in recreational or moderately trained subjects.

## Author Contributions

All authors contributed to the study conception, analysis and interpretation, drafting the paper, and gave their final approval of the manuscript.

## Conflict of Interest Statement

MGi and SC were employed by the Salomon company (France). The remaining authors declare that the research was conducted in the absence of any commercial or financial relationships that could be construed as a potential conflict of interest.
